# Arabidopsis Motif Scanner

**DOI:** 10.1186/s12859-016-0896-x

**Published:** 2016-01-27

**Authors:** Giovanni Mele

**Affiliations:** Institute of Agricultural Biology and Biotechnology (IBBA), National Council of Research (CNR), Via Salaria Km. 29.300, Monterotondo Scalo (Roma), 00015 Italy

**Keywords:** Arabidopsis, *cis*-elements, Binding motif, Gene network, Next Generation Sequencing, DNA Chip Array, Gene expression

## Abstract

**Background:**

The major mechanism driving cellular differentiation and organism development is the regulation of gene expression. *Cis*-acting enhancers and silencers have key roles in controlling gene transcription. The genomic era allowed the transition from single gene analysis to the investigation of full transcriptomes. This transition increased the complexity of the analyses and the difficulty in the interpretation of the results. In this context, there is demand for new tools aimed at the creation of gene networks that can facilitate the interpretation of Next Generation Sequencing (NGS) data.

**Results:**

Arabidopsis Motif Scanner (AMS) is a Windows application that runs on local computers. It was developed to build gene networks by identifying the positions of *cis*-regulatory elements in the model plant *Arabidopsis thaliana* and by providing an easy interface to assess and evaluate gene relationships. Its major innovative feature is to combine the *cis*-regulatory element positions, NGS and DNA Chip Arrays expression data, Arabidopsis annotations and gene interactions for the identification of gene networks regulated by transcription factors. In studies focused on transcription factors function, the software uses the expression data and binding site motifs in the regulative gene regions to predict direct target genes. Additionally, AMS utilizes DNA-protein and protein-protein interaction data to facilitate the identification of the metabolic pathways regulated by the transcription factor of interest.

**Conclusions:**

Arabidopsis Motif Scanner is a new tool that helps researchers to unravel gene relations and functions. In fact, it facilitates studies focused on the effects and the impact that transcription factors have on the transcriptome by correlating the position of cis-acting elements, gene expression data and interactions.

## Background

The recent advent of the genomic era, first with the DNA Chip Arrays then with Next Generation Sequencing (NGS) technologies, revolutionized the classical view that divided the genome into two entities: one comprising genes and their regulatory regions responsible for encoding messenger RNA translated in turn into protein; and one comprising “junk DNA” with unknown and consequently nonessential function [1]. By NGS, it was possible to identify that non-coding transcripts (ncRNA), which originate from intergenic sequences previously defined as junk, far exceed those of protein-coding genes [2]. This led to the discovery of novel layers of complexity in gene organization and expression highlighting the extreme versatility of genomes [3, 4]. Moreover, the genomic era has radically modified the entire field of biology by changing the approach and the challenges that biologists have to face [5–8]. Indeed, the advent of NGS technologies has enabled researchers to move from single gene analysis to the investigation of full transcriptomes. Although this transition allows one to have a global view on the gene expression changes, it came at a price. On one side, the enormous amount of data increased the complexity of the analyses and of the management of information; on the other side, it increased the difficulties in the interpretation of the results obtained. In the near future the major challenge in software development will be platforms that can best integrate the NGS results to facilitate data interpretation. This new generation of programs will be considered successful when they will provide an extensive view on gene interactions and molecular pathways that can help the comprehension of gene relations and functions.

Cellular differentiation and organism development are governed by precise gene expression patterns. The diverse expression patterns in the different cells are established by the coordinated action of intergenic, as well as intragenic, *cis*-acting enhancers and silencers known as *cis*-regulatory elements [9–11]. *Cis*-acting elements such as core and proximal promoter elements are typically restricted to within a couple of hundred base pairs from transcriptional start sites and regulate genes in their immediate vicinity. In contrast, distal *cis*-elements are usually located at >1 kb and in some cases up to 1 Mb in either direction from a transcription start site. Functional DNA sequences change at a lower rate over evolutionary time than sequences without function [12, 13]. Consequently, *cis*-regulatory elements tend to be conserved, whereas functionless sequences are randomized by substitution, lost by conversion, or deleted entirely. In this context, genome-wide studies of *cis*-regulatory elements become the key path to build metabolic pathways and gene networks for a better comprehension of gene relations and transcription factors function. In fact, the first step to unravel the function of a transcription factor is the identification of the *cis*-regulatory element that it binds and the target genes under its control. Subsequently, the clusterization of the target genes in networks allows the identification of the metabolic pathways regulated by the transcription factor of interest and consequently of its function.

To date, several different platforms for expression data analysis and management have been developed; however, there is a demand for software for Arabidopsis data interpretation. Most of the available promoter analysis software focuses on the presence of well-characterized *cis*-acting elements in a single user provided promoter region. Alternatively, they scan the Arabidopsis genome and provide a list of loci where the *cis*-acting element is present. AMS facilitates transcription factors function identification by implementing data interpretation. In fact, AMS allows the search of *cis*-acting elements, the organization of the differentially expressed target gene data and the identification of gene networks.

## Implementation

**Fig. 1:**
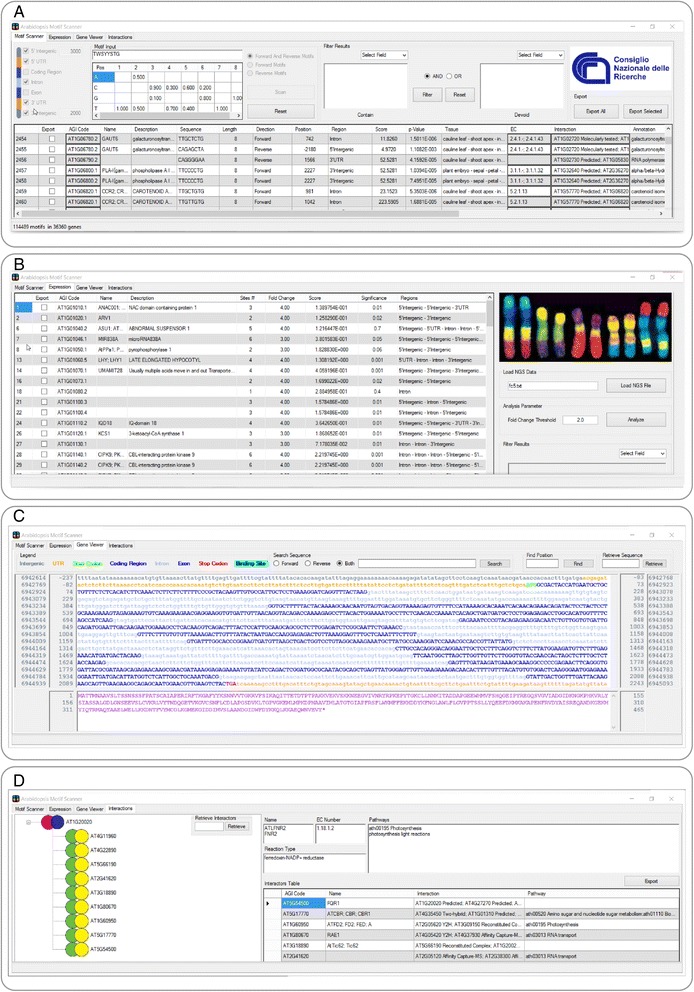
Arabidopsis Motif Scanner Tabs. **a** *Motif Scanner* Tab. **b** Expression Tab. **c** *Gene Viewer* Tab. **d** *Interactions* Tab

Arabidopsis Motif Scanner (AMS) executable is freely available on the web page of the Institute of Agricultural Biology and Biotechnology of National Council of Research (http://www.ibba.mlib.cnr.it/Arabidopsis_Motif_Scanner.html) and at SourceForge open-source repository (http://sourceforge.net/projects/arabidopsismotifscanner/files/?source=navbar). This software was developed in C# language and was designed to be fully compatible with Windows 7, 8 and 10 environments. Arabidopsis Motif Scanner is a user-friendly application developed for the Windows environment to be fully compatible with the Illumina NGS and the Affymetrix Gene Chip platforms. Arabidopsis Motif Scanner GUI consists of one window with four tabs: *Motif Scanner, Expression*, *Gene Viewer* and *Interactions* (Fig. 1).

### Motif scanner Tab

The Arabidopsis Motif Scanner starts up by loading the database and displays the *Motif Scanner* tab (Fig. 1a). The tab consists of an upper setting mask dedicated to the input of working parameters and a lower mask that shows the results.

The setting mask includes four panels (from left to right): *chromosome region selection*; *motif input*; *filter function* and *data export*. In the *chromosome region selection* panel, the tick boxes allow the selection of chromosome regions that will be included in the analysis. The rectangular boxes, which flank the 3’ and 5’ intergenic tick boxes, provide analysis flexibility by allowing the user to select the intergenic sequence length. If the intergenic length boxes are left blank, the software considers as intergenic the regions between genes. In the case that intergenic sequences are shorter than the user input, the intergenic regions will be considered up to the flanking coding sequence.

The *motif input* panel contains the user input sequence box and allows the search for the motif in forward, reverse or both orientations. The software accepts standard nucleotide degeneration (Table 1) and an editable Position-specific Weight Matrix (PWM) allows a refined and flexible search. The PWM expresses the probability that the transcription factor of interest binds the motif considered. For transcription factors with a known binding motif sequence the PWM matrix can be easily retrieved from the literature (PWM sequence logo), while for transcription factors with unknown binding motif sequence the PWM matrix is obtained experimentally by the Selection And Amplification Binding (SAAB) assay.

**Table 1:** Degeneration Table. The first and third column report the character assigned to the degeneration. In the second and forth columns show the correspondent nucleotides

Deg	Nuc	Deg	Nuc
R	A, G	B	C, G, T
Y	C, T	D	A, G, T
S	G, C	H	A, C, T
W	A, T	V	A, C, G
K	G, T	N	A, G, C, T
M	A, C		

To obtain a reasonable number of output hits for the subsequent data interpretation, a motif of at least 7 bp long is suggested. To refine the outputs, a filter can be set; the selective parameters are typed in the *Contain* and *Devoid* windows to function in parallel or in exclusive way (using the “and/or” radio buttons). The *data export* panel consents the export of results in a tab delimited text file. The tickable cells of the export column in the results table allows a selective export of the data of interest.

The lower mask reports a column-sortable table which visualizes AGI Code, Name of the gene, Description of gene, Sequence of the targeted motif, Length of the targeted motif, Region within the gene that holds the motif, Direction of the motif, Position with respect to the start codon, Motif Score, *p*-Value, Significance, EC Number (unique number assigned to a enzymatic reaction), Interaction of the gene product and the Annotation. Moreover, the AGI Code, EC and Interaction cells in the results table are clickable and respectively display gene organization inclusive of the binding motif positions, pathways in which the gene is involved and its protein interactions. The table further reports all matching motifs for all the different splicing forms. Finally, the total number of motifs appears at the bottom left corner.

Each Motif Score (*MS*) is calculated based on *observed:expected* frequency ratios (O/E) and takes into account the *PWM* specific motif value. Specifically, each *MS* is computed as:

$$ MS={V}_{PWM}*\ {B}_O\ *\  \ln \left(\raisebox{1ex}{${B}_O$}\!\left/ \!\raisebox{-1ex}{${B}_E$}\right.\right) $$

Where V_PWM_ expresses the probability that the transcription factor of interest binds that exact motif sequence considered and it is calculated on the PWM matrix created by the user. *B*_*O*_ and *B*_*E*_ represent the number of Binding sites Observed and Expected for the motif of interest respectively. *B*_*E*_ is calculated as:

$$ {B}_E={M}_P*T $$

*M*_*p*_ is the probability to observe the motif and depends on the motif sequence composition and the nucleotide frequencies in the genome (for Arabidopsis A,T = 0.32 and C,G = 0.18). *M*_*p*_ is calculated as the frequency of each nucleotide that constitutes the motif (e.g. M_P_ for ATCCG = 0.32x0.32x0.18x0.18x0.18). *T* represents the total number of possible matching positions for the input motif across both strands of the genome and it is calculated as:

$$ T=2*\left({R}_L-{M}_L+1\right) $$

Where *2* accounts for two strands. *R*_*L*_ is the length of the DNA region where the motif occurs. *M*_*L*_ is the motif length.

The *p*-Value for each motif represents the probability of obtaining at least n motifs in the sequence and it is calculated by the cumulative binomial distribution.

$$ \mathrm{p}-\mathrm{Value}=1-\sum_{i=0}^{n-1}\left(\begin{array}{c}T\\ {}i\end{array}\right)\ast {\left({M}_P\right)}^i\ast {\left(1-{M}_P\right)}^{T-i} $$

Where *n* is the number of motifs in the sequence. *M*_*p*_ is the probability to observe the motif and *T* represents the total number of possible matching positions.

### Expression Tab

The Expression tab (Fig. 1b) allows the integration between *cis*-acting element positions and NGS or DNA Chip Array expression data. In the upper right section of the panel, a tab-delimited file containing the AGI code and the correspondent fold Change can be loaded. The Fold Change Threshold (FCT) box allows the user to set the best FCT for the analysis. The lower right box permits filtering the result. In the left section, a column-sortable table visualizes the AGI code Name, Description, Number of Sites, Fold Change, Regions, Binding Score and Significance for each gene.

The Binding Score (BS) is computed as:

$$ BS={\displaystyle \sum_{i=1}^n}{V}_{PWM}*\ {B}_{FC\  spec}*\  \ln \left(\frac{\raisebox{1ex}{${B}_{FC\  Spec}$}\!\left/ \!\raisebox{-1ex}{${B}_{Tot\  Spec}$}\right.}{\raisebox{1ex}{${B}_{FC}$}\!\left/ \!\raisebox{-1ex}{${B}_{Tot}$}\right.}\right) $$

Where *n* symbolizes the number of binding motifs present in each user-defined locus (genomic regions selected for the analysis). V_PWM_ expresses the probability that the transcription factor of interest binds that exact motif sequence considered and it depends on the PWM matrix created by the user. *B*_*FC*_ and *B*_*FC Spec*_ represent the occurrence of binding sites above the fold change threshold for all the motifs derived from the PWM and for one specific motif, respectively. The fold change threshold is set by the user in the FCT box in the “Expression Tab” and depends only on the expression data quality. Finally, the *B*_*tot*_ and *B*_*tot Spec*_ indicate the total motif number occurring in the genome for all the motifs considered derived from the PWM and for one specific motif, respectively. Moreover, the chi-squared test is used to determine whether there is a significant difference between the number of genes that contain binding sites and the number of genes that both are differentially expressed and contain binding sites for all the motifs derived from the PWM and for the one specific motif considered.

In the studies focused on the function of a transcription factor, the comparison between wild-type sample and mutant that over or under express the transcription factor of interest is a key step for the identification of the target genes. In this type of studies it is informative to distinguish between target genes which are directly bound by the transcription factor that alters their expression (primary targets) from genes with a mutated expression due to secondary effects (secondary targets). In AMS, the possibility to combine binding site positions with changes of expression (expressed as fold change) allows the discrimination between primary and secondary target genes.

### Gene viewer Tab

The *Gene Viewer* tab (Fig. 1c) shows the gene organization by either clicking on the AGI code box of the *Motif Scanner* tab or by typing the AGI code in the *Retrieve Sequence Box* (top right). The upper and bottom sections include the gene structure and the protein sequence, respectively. The different gene regions appear colored according to the legend (upper left corner). Both left and right external columns report the absolute nucleotide position in the chromosome, while the internal columns include the positions with respect to the ATG codon start. The user can search for specific nucleotide sequences or positions by typing the *Search Sequence* and *Fine Position* functions. In the bottom section, the protein translation is displayed and is flanked by two columns, which report the aminoacid position with respect to the initial Methionine. Finally, the length and position of a selected sequence appear in the lower left corner.

### Interactions Tab

The *Interactions* tab is displayed (Fig. 1d) after clicking on either EC or Interaction cells of the *Motif Scanner* tab. A tree view of the interactions appears in a window on the left side. Upon selection, the yellow and green interaction symbol turns blue and fuchsia showing the biochemically tested and computational assigned interactors of the gene of interest. Concerning the biochemically tested interactions, the AMS database contains information of protein-protein interaction, Protein Complementation Assay, Far Western Blotting, Pull Down, biochemical activity, Affinity Capture-Western, Yeast Two-Hybrid, Pull-Down Assay, Affinity Capture-MS, Co-Immunoprecipitation,in vitro Binding Assay, Split-Reporter Assay and phenotypic suppression and enhancement.

The right side of the *Interactions* tab consists of two panels, the top one reports the Name, EC number, Reaction Type and Pathways referred to the targeted gene, while, the bottom panel provides more information on the interactors, including the AGI Code, Name, Interactions and Pathway of the interactors. The tree view and the interactors table are synchronized for an easy consultation.

### Database

The database behind the Arabidopsis Motif Scanner was built using several different sources. Arabidopsis genome organization and annotations was derived from “The Arabidopsis Information Resource” (TAIR) consortium (http://www.arabidopsis.org) and elaborated to best fit the requirements of the software. Biologically tested and computational derived interactions were retrieved from: 1) AtPID database (Jian C., *et al*. 2007) (http://www.megabionet.org/atpid/webfile); 2) the database published by Jane G. L. (Jane G. L., *et al* (2007); 3) the Plant Interactome Database resulted from a collaboration between the Salk Institute (http://signal.salk.edu/interactome/index2.html) and the Center for Cancer Systems Biology (http://interactome.dfci.harvard.edu/A_thaliana/index.php); 4) BioGRID (http://www.thebiogrid.org/); 5) IntAct of EMBL-EBI (http://www.ebi.ac.uk/intact/); 6) BIND (http://bind.ca/); 7) TAIR.

## Results and discussion

The majority of the web interfaces available on the net are developed to perform analyses of *cis*-acting elements on human and animal genomes. Moreover, several of them are commercial platforms and analyses are typically expensive. As for the plant kingdom, the web interfaces were developed for Arabidopsis since, as model plant, it has the best-annotated genome. Web interfaces such as PLACE (https://sogo.dna.affrc.go.jp/cgi-bin/sogo.cgi?lang=en&pj=640&action=page&page=newplace) were developed based on non-holistic criteria, in other words, the analyses can be performed only for well-known *cis*-acting elements and are restricted to single promoters. On the contrary, the Arabidopsis Motif Scanner was developed to identify the positions of new and unknown *cis*-acting elements in the entire genome by using an easy-to-use GUI. The AMS software shows a few similarities with genome-scale-DNA-pattern match of Regulatory Sequence Analysis Tools (RSAT) (http://floresta.eead.csic.es/rsat/) and PatMatch of The Arabidopsis Information Resource (TAIR) (http://www.arabidopsis.org/cgi-bin/patmatch/nph-patmatch.pl) web-based GUIs. Differently from those, AMS provides gene annotation and information on gene interactions, which are essential for the identification and construction of gene networks and integration with NGS expression data for the comprehension of new gene relations and functions.

Arabidopsis Motif Scanner is more flexible than PatMatch and RSAT in the choices of the genomic regions. For instance, Arabidopsis Motif Scanner allows choosing any combination of genomic regions while PatMatch has some limitation not allowing analyzing upstream and downstream regions in the same run. Concerning RSAT, it does not permit searches in multiple regions. The AMS flexibility allows identifying motifs falling across two contiguous regions that it is not possible to be identified with both PatMatch and RSAT. Moreover, PatMatch poses limits on the intergenic regions length (500 or 1000 or 3000 bp) and does not take into account if the intergenic regions are shorter because of the presence of coding sequences of flanking genes. These limitations are overcome in AMS that consider intergenic region the portion of sequence between two coding sequences. Finally, AMS offers the option to use PWM for the input motif differently from PatMatch and RSAT.

Most genes encode multiple transcripts by alternative promoters, alternative splicing, or alternative polyadenylation [14–18]. The combinatorial mechanism of alternative splicing increases the coding potential of the genome by allowing the synthesis of multiple protein isoforms with different—even antagonistic—functions from a single gene [19]. AMS offers the advantage to analyze the motif occurrence in multivariate splicing forms, providing information on why differentially spliced transcripts are found in the diverse tissues and organs. Moreover, the Gene Viewer Tab is an easy tool to visualize motif positions and organization for the genes of interest. Contrarily to PatMatch and RSAT, AMS reports the motif in the 5’ regions as “negative” positions with respect to the ATG start. One peculiar feature of AMS is the possibility to combine *cis*-acting element search and Next Generation Sequencing data for a better comprehension of gene relations and functions.

AMS becomes particularly useful for the studies of transcription factor (TF) function. To this aim, two aspects must be considered to an effective result. First, it is mandatory to identify the primary targeted genes of the TF and their respective functions. Second, it is necessary to detect transcriptome differences between wild-type plants versus mutant, which is affected in a given TF. AMS output consists of a gene list that match motif occurrence and intensity of differential expression allowing a significant selection of putative primary targets. The list of this primary targets combined with gene annotations and interaction further provides the information on molecular pathways in which a TF exerts functions.

## Conclusion

Arabidopsis Motif Scanner is a powerful user-friendly tool that runs on local computers allowing correlation of genomic–wide searches for *cis*-acting element position and NGS or DNA Chip Array expression data. The great advantage of the software, that distinguishes it from other web interfaces, is the presence of a database that provides annotations and gene interactions for the hits. When combined with expression data, this software enhances the interpretation of NGS and DNA Chip Array results and allows the discovery of new gene relations and functions. Moreover, Arabidopsis Motif Scanner can be considered an effective tool to identify new functions of transcription factors. In fact, the genome-wide screen of transcription factors binding motifs provides valuable information on the probable target genes and consequently on the metabolic pathways in which the transcription factor of interest is involved.

### Availability and requirements

Project name: Arabidopsis Motif Scanner

Project home page: http://www.ibba.mlib.cnr.it/Arabidopsis_Motif_Scanner.html

Operating system(s): Windows

Programming language: C#

Any restrictions to use by non-academics: license needed: GNU General Public License version 3.0 (GPLv3)
